# Systematic evaluation of pigment-based whole-cell lead biosensors: challenges in genetic circuit engineering and critical considerations for background noise control

**DOI:** 10.3389/fbioe.2025.1744651

**Published:** 2026-01-07

**Authors:** Yan Guo, Li-dan Deng, Wen-wu Gong, Juan Zhang, Chang-ye Hui

**Affiliations:** 1 Shenzhen Prevention and Treatment Center for Occupational Diseases, Shenzhen, China; 2 Department of Epidemiology and Biostatistics, School of Public Health, Jilin University, Changchun, China

**Keywords:** background noise control, genetic circuit engineering, lead (II) detection, pigment signal, whole-cell biosensor

## Abstract

**Introduction:**

Despite advances in whole-cell biosensors for Pb(II) detection, background leakage and limited dynamic range hinder field deployment. This study systematically evaluates four diverse *pbr* operons and optimizes genetic circuit architectures to develop high-performance pigment-based Pb(II) biosensors.

**Methods:**

pbr operons from *Cupriavidus metallidurans, Pseudomonas aeruginosa*, and *Klebsiella pneumoniae* were engineered into *E. coli* TOP10 using decoupled violacein reporter circuits. Five optimization strategies were tested: transcriptional terminators, graded regulator expression (P_302_-P_J23119_ promoters), dual-gene copies, hybrid MerR-PbrR regulators, and circuit architecture variants. All assays used triplicate replicates (n = 3) across 8 time points and 12-15 Pb(II) concentrations.

**Results:**

The pLVPK-derived pKp-DV construct (PbrR.Kp) achieved optimal performance: LOD of 0.0008 μM, dynamic range 0.0061-50 μM, and high Pb(II) specificity. Transcription terminator insertion further reduced LOD to 0.0002 μM and background noise by 60%. However, regulator dosage and hybrid circuits showed minimal improvement, indicating limited synergistic benefits.

**Discussion:**

PbrR.Kp selection and transcriptional insulation are key strategies for reducing leakage. Hybrid circuits require more sophisticated designs. Future work should focus on chromosomal integration and environmental matrix validation to ensure the development of robust, field-ready sensors.

## Graphical Abstract

**Figure:**
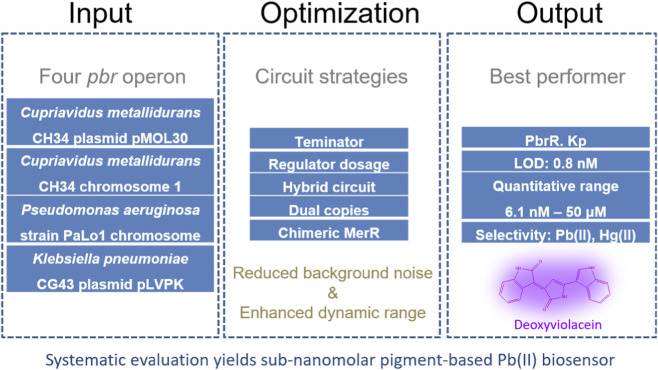

## Introduction

Whole-cell biosensing technology has emerged as a powerful tool for environmental monitoring, offering distinct advantages over traditional instrumental methods, which require sophisticated equipment, sample pre-treatment, and well-trained personnel (Liu et al., 2022). Whole-cell biosensors provide a more accessible and cost-effective alternative. They harness the innate biological mechanisms of microorganisms to detect and respond to specific environmental contaminants, such as lead ions (Pb(II)), with sensitivity and selectivity (Hui et al., 2023). The simplicity of these biosensors allows for rapid, real-time analysis with minimal equipment, making them ideal for on-site monitoring and large-scale screening.

Various types have been explored and utilized in the context of biosensing signals. These include bioluminescent, fluorescent, and colorimetric signals, each with unique characteristics (Hui et al., 2024a; Hui et al., 2024b). Among them, colorimetric signals provide a visible and cost-effective means of detection that does not necessitate complex instrumentation. Recent advances in non-pigment-based lead biosensors have introduced electrochemical methods utilizing whole bacterial cells (Tataru et al., 2023; Liu et al., 2025) and nanozyme-based colorimetric platforms (Qu et al., 2023; Wen et al., 2024; Yu et al., 2024; Chen et al., 2026), offering alternative detection modalities with rapid response times. However, pigment-based biosensors, especially those that harness the natural biosynthetic pathways of microorganisms, produce various colorants in response to the presence of heavy metals, thereby providing a visual readout of contamination levels (Nemer et al., 2024). The use of pigments such as violacein and its derivatives allows for the creation of biosensors that are not only sensitive but also cost-effective and easy to implement in monitoring various environmental samples, including freshwater, seawater, soil extract, and even biological samples (Hui C. Y. et al., 2022; Guo et al., 2024b; Hu et al., 2024; Ma et al., 2024; Wu et al., 2024).

Bacterial whole-cell biosensors consist of a sensing and a reporter module. The sensory module is typically genetically modified using natural metal-resistant operons as templates, which trigger pigment production upon binding with intracellular metal ions (Hui et al., 2021b; Liu et al., 2022; Hui et al., 2024a). The specificity of bacterial sensors requires the sensory module to bind only to the target metals. However, specific protein-metal interactions are inherently complex due to the lack of distinguishable physicochemical features of metal ions (He et al., 2021).

To address the challenges above, genetic engineering has facilitated the tailoring of biosensors, thereby enhancing their specificity and sensitivity towards targeted metals. The *pbr* operon, first identified in *Cupriavidus metallidurans* CH34, represents a pivotal genetic element that encodes a multifaceted resistance mechanism against Pb(II), inclusive of transport, efflux, sequestration, biomineralization, and precipitation (Borremans et al., 2001). The process of molecular evolution and genetic mutation, facilitated by horizontal gene transfer, has led to the emergence of diverse PbrR homologs and their associated operons, which are readily identifiable in genomic databases (Hui et al., 2023). Despite this, the application of these diverse *pbr* operons in whole-cell biosensing has yet to be thoroughly explored.

In this study, we have harnessed the principles of synthetic biology and metabolic engineering to develop whole-cell biosensors utilizing the purple pigment deoxyviolacein (DV) as a visual reporter. We selected four distinct *pbr* operons from *Cupriavidus metallidurans* CH34 plasmid pMOL30, *Cupriavidus metallidurans* CH34 chromosome 1, *Pseudomonas aeruginosa* strain PaLo1 chromosome, and *Klebsiella pneumoniae* CG43 plasmid pLVPK as templates to engineer bacteria with enhanced biosensing capabilities. Through a systematic comparison of their biosensing performance, we evaluated vital parameters, including background noise, which is indicative of the stringent transcriptional regulation of *pbr* elements; selectivity, which reflects the metal-binding specificity of PbrR homologs; and dose-response relationships, which indicate the biosensors' practical applicability.

This study presents a comprehensive evaluation of four distinct *pbr* operons from various bacterial species, systematically comparing their biosensing performance metrics. Among these, the *pbr* operon from *Klebsiella pneumoniae* CG43 plasmid pLVPK (PbrR.Kp) emerged as the most effective for Pb(II) detection, achieving a low detection limit (0.0008 µM) compared to the poorest performer (PbrR.Cm, 0.0977 µM), a broad dynamic range (0.0061–50 µM), and the fastest response time (3 h). We explored five genetic circuit engineering strategies to optimize biosensing performance, including the insertion of transcriptional terminators, graded regulator dosage control, dual-gene copy modulation, and a hybrid MerR-PbrR regulator fusion. This analysis revealed that decoupled circuits expanded the detection range 10-fold but increased the background noise by a factor of 3. In contrast, terminators reduced LOD by 4-fold without affecting range, yet hybrid constructs showed no synergistic benefits. Our findings confirm that the selection of PbrR homologs critically governs the specificity of PbrR.Kp showed the highest Pb(II) selectivity over Cd(II) and Zn(II) due to its unique metal-binding pocket architecture (Guo et al., 2025a; Hui, 2025). In contrast, circuit architecture determines the balance between sensitivity and background leakage. Future work will focus on reducing background noise and enhancing sensitivity by exploring higher-threshold pigments such as indigoidine (Hui et al., 2021a; Wu et al., 2025), indigo (Guo et al., 2024a), or pyomelanin (Tang et al., 2025), and systematically validating performance in complex environmental matrices.

## Materials and methods

### Bacterial strains, plasmids, and culture conditions

The vectors utilized in this study are detailed in Table 1, and the DNA sequence of core elements is listed in Supplementary Table S1. *E. coli* TOP10 was the host cell for cloning procedures and constructing whole-cell biosensors. All engineered bacterial strains were cultured in Luria–Bertani (LB) broth, prepared with 10 g/L tryptone, 5 g/L yeast extract, and 10 g/L NaCl, and supplemented with 50 μg/mL ampicillin to maintain antibiotic selection. Stock solutions of heavy metal salts, including zinc sulfate, lead nitrate, cadmium chloride, and mercury chloride, were freshly prepared using analytical-grade chemicals and distilled water. All DNA fragments required for this study were custom-synthesized by Sangon Biotech (Shanghai, China).

**TABLE 1:** Bacterial strain and plasmids used in this study.

Strains and plasmids	Genotypes or description	Reference
*E. coli* strain		
TOP10	F^−^ Φ80*lac*ZΔM15 Δ*lac*X74 *rec*A1	Tiangen
Plasmids		
pPb-vioABCE	The *vioABCE* gene cluster fused downstream of a natural lead-resistant promoter from *Klebsiella pneumoniae* CG43 plasmid pLVPK	Hui C. Y. et al. (2022)
pPmer-vioABCE	The *vioABCE* gene cluster was fused downstream of a natural mercury-resistant promoter from *E. coli* Tn21	Ma et al. (2024)
pCm-DV	The *vioABCE* gene cluster fused downstream of the designed Pb(II)-responsive element from *Cupriavidus metallidurans* CH34 plasmid pMOL30	This study
pCmc-DV	The *vioABCE* gene cluster fused downstream of the designed Pb(II)-responsive element from *Cupriavidus metallidurans* CH34 chromosome 1	This study
pPa-DV	The *vioABCE* gene cluster was fused downstream of the designed Pb(II)-responsive element from *the Pseudomonas aeruginosa* strain PaLo1 chromosome	This study
pKp-DV	The *vioABCE* gene cluster was fused downstream of the designed Pb(II)-responsive element from *the Klebsiella pneumoniae* CG43 plasmid pLVPK	This study
pTer-Kp-DV	pKp-DV derivative with a *rrn*B terminator inserted upstream of designed Pb(II)-responsive element from *Klebsiella pneumoniae* CG43 plasmid pLVPK	This study
p302-Kp-DV	The pKp-DV derivative, in which the expression of regulator PbrR from *Klebsiella pneumoniae* CG43 is under the control of the constitutive P_302_ promoter	This study
p406-Kp-DV	The pKp-DV derivative, in which the expression of regulator PbrR from *Klebsiella pneumoniae* CG43 is under the control of the constitutive P_406_ promoter	This study
p479-Kp-DV	The pKp-DV derivative, in which the expression of regulator PbrR from *Klebsiella pneumoniae* CG43 is under the control of the constitutive P_479_ promoter	This study
p535-Kp-DV	The pKp-DV derivative, in which the expression of regulator PbrR from *Klebsiella pneumoniae* CG43 is under the control of the constitutive P_535_ promoter	This study
p637-Kp-DV	The pKp-DV derivative, in which the expression of regulator PbrR from *Klebsiella pneumoniae* CG43 is under the control of the constitutive P_637_ promoter	This study
p699-Kp-DV	The pKp-DV derivative expresses the regulator PbrR from *Klebsiella pneumoniae* CG43 under the control of the constitutive P_699_ promoter	This study
pJ23119-Kp-DV	The pKp-DV derivative, in which the expression of regulator PbrR from *Klebsiella pneumoniae* CG43 is under the control of the constitutive P_J23119_ promoter	This study
pNAT-MP-DV	pPmer-vioABCE derivative with the metal-binding domain of MerR substituted with that of PbrR	This study
pCON-MP-DV	pNAT-MP-DV derivative with chimeric MerR-PbrR regulator under the control of constitutive P_*cueR*_ promoter	This study
pKp-2P-DV	pKp-DV derivative with an extra *pbrR* ORF inserted in front of the divergent *pbr* promoter	This study

### Design rationale for genetic circuit configurations

The four native *pbr* operons were selected to capture natural genetic diversity arising from horizontal gene transfer, which may encode PbrR variants with altered metal-binding cavities and promoter stringency (Hui et al., 2023). Hybrid *pbr*-*mer* circuits test the hypothesis that fusing MerR’s high-affinity DNA-binding domain with PbrR’s Pb(II)-selective metal-binding domain could reduce basal leakage—a known limitation of native *pbr* promoters (Fang and Zhang, 2022). Decoupled architectures separate regulatory and reporter functions, enabling the independent optimization of regulator dosage to minimize background expression while expanding the dynamic range, a fundamental principle for reducing genetic circuit crosstalk (Ling et al., 2025; Ou et al., 2025).

Promoter strength modulates PbrR protein abundance. Regulator expression level controls promoter occupancy kinetics (Liu et al., 2024). The operon structure has a critical effect on transcriptional interference. Natural divergent promoters are prone to read-through transcription, which increases the basal signal. In contrast, the insertion of *rrn*B terminators in decoupled circuits reduces background but may increase response latency due to the separate transcriptional units (Guo M. et al., 2019).

### Plasmids construction

The origins of the four *pbr* operons, derived from various bacterial species utilized in this study, are comprehensively listed in Table 2. These operons were strategically selected to maximize genetic diversity: (1) *Cupriavidus metallidurans* pMOL30 plasmid (pCm) as the archetypal system, (2) chromosomal *C. metallidurans* operon (pCmc) with a truncated promoter, (3) uncharacterized *Pseudomonas aeruginosa* PaLo1 (pPa) with an extended intergenic region, and (4) high-performance *Klebsiella pneumoniae* pLVPK (pKp) based on prior success. Unique features include full-length vs. compact promoters and distinct regulatory architectures. The PbrR.Pa homolog was identified through BLAST analysis, which revealed significant homology with PbrR.Cmc. However, PbrR.Pa is distinguished by a more extended divergent *pbr* promoter, similar in length to those of PbrR.Cm and PbrR.Kp. The pLVPK-derived PbrR.Kp was selected for hybridization/decoupling experiments because Scheme I screening demonstrated superior LOD and dynamic range, making it the most promising template. The plasmids pPb-vioABCE and pPmer-vioABCE, previously developed for biosensing Pb(II) and Hg(II), respectively, employ naturally coupled circuits. Both plasmids utilize the *vioABCE* gene cluster for the biosynthesis of purple deoxyviolacein (DV), which serves as a visual and colorimetric signal for biosensing.

**TABLE 2:** The origin of the lead resistance operon redesigned in this study.

Origin	GenBank accession	Screening constructs	Ref
*Cupriavidus metallidurans* CH34 plasmid pMOL30	CP000354.2	pCm-DV	Wei et al. (2014)
*Cupriavidus metallidurans* CH34 chromosome 1	CP000352.1	pCmc-DV	Huang et al. (2016)
*Pseudomonas aeruginosa* strain PaLo1 chromosome	CP075849.1	pPa-DV	\
*Klebsiella pneumoniae* CG43 plasmid pLVPK	AY378100	pKp-DV	Zhu et al. (2023)

Newly constructed plasmids pCm-DV, pCmc-DV, pPa-DV, and pKp-DV feature non-coupled circuits, with their respective PbrR homologs under the regulation of the constitutive promoter P_*ceuR*_. The plasmid pTer-Kp-DV was engineered by introducing a transcription terminator upstream of the divergent *pbr* promoter of pKp-DV.

A series of constructs, p302-Kp-DV, p406-Kp-DV, p479-Kp-DV, p535-Kp-DV, p637-Kp-DV, p699-Kp-DV, and pJ23119-Kp-DV, were assembled using non-coupled circuits. Within this series, increasingly stronger constitutive promoters controlled the PbrR protein from *Klebsiella pneumoniae* CG43 (PbrR.Kp). An additional copy of PbrR.Kp was inserted downstream of the divergent *pbr* promoter of pKp-DV, resulting in the plasmid pKp-2P-DV.

A chimeric regulator, PbrRMerR, was designed to replace the MerR regulator in pPmer-vioABCE, yielding the plasmid pNAT-MP-DV, which employs a natural coupled circuit. Furthermore, a constitutive promoter P_*ceuR*_ was placed upstream of the PbrRMerR-encoding gene to create pCON-MP-DV, utilizing a non-coupled circuit.

All biosensing constructs were chemically transformed into *E. coli* TOP10 to assemble whole-cell bacterial biosensors for the following studies. The engineered bacteria were aerobically incubated in 15 mL bioreactor tubes equipped with vented lids, allowing for gas exchange (Jet Bioreactor Tubes, Guangzhou, China). The incubation was conducted at a controlled temperature of 37 °C. The culture was stirred at 250 revolutions per minute to ensure homogeneous mixing and optimal aeration conditions.

### Time-response assay

The engineered bacteria were initially cultured overnight to achieve activation. Subsequently, the culture was diluted to a 1:100 ratio in fresh LB medium to initiate the time-response assay. The diluted bacterial suspension was then exposed to final concentrations of 0 and 25 μM Pb(II) for 7 h. The assay monitored bacterial growth and the DV signal at 1-h intervals to evaluate the response to Pb(II) exposure. The induction coefficient was calculated as the ratio of the DV signal in the Pb(II)-supplemented LB medium to that in the control LB medium without Pb(II) supplementation to quantify the effect of Pb(II) on the DV signal.

### Selectivity assay

Activated engineered bacteria were diluted to a 1:100 ratio in fresh LB broth. The cultures were then exposed to various concentrations of Pb(II), Cd(II), Zn(II), and Hg(II) to induce the possible response. The concentrations used were 0, 1.5625, 3.125, 6.25, 12.5, 25, 50, and 100 μM, employing a double-dilution method (Hui et al., 2021c) to increase the metal ion concentration gradually. The exposure times varied depending on the plasmid construct used: 4 h for the strains TOP10/pCm-DV and TOP10/pPa-DV, and 3 h for the strains TOP10/pKp-DV and TOP10/pCmc-DV. Following the exposure period, the bacterial density and the DV signal were measured to assess the selectivity of the engineered bacteria towards the different metal ions.

### Dose-response assay

Activated engineered bacteria were diluted to a 1:100 ratio in fresh LB broth to standardize the assay conditions. The cultures were then subjected to a range of Pb(II) concentrations (0–800 µM) to evaluate the dose-dependent response of the engineered bacteria to Pb(II) exposure, with specific ranges tailored to each biosensor construct. The induction with Pb(II) was performed for various durations as described above. Following the exposure, we measured the bacterial density and the DV signal and drew dose-response curves.

### Determination of bacterial density and pigment signals

An aliquot of 100 μL from the bacterial samples was carefully pipetted into each well of a 96-well plate. The bacterial suspension’s optical density (OD) was then measured at a wavelength of 600 nm using a microplate reader (BioTek Epoch, Winooski, VT) to assess the cell density. The remaining 900 μL bacterial liquid was transferred into a centrifuge tube and mixed with 360 μL of n-butanol. This mixture was vortexed to facilitate the extraction of DV and then subjected to centrifugation to separate the phases. Subsequently, 100 μL of the supernatant was carefully transferred to a new 96-well plate. The absorbance of the DV in the supernatant was measured at a wavelength of 570 nm, which is characteristic of the quantification of DV.

### Statistical analysis

All assays were independently repeated at least three times. Data normality was assessed using the Shapiro–Wilk test. These variables are reported as the mean ± standard deviation (SD) to measure central tendency and dispersion. The Independent Samples t-test was utilized to compare the differences between the two independent groups. For comparisons of a single sample to a known population mean, the One-Sample t-test was employed. Statistical significance was defined as a two-tailed P-value less than 0.05. The limit of detection (LOD) was determined using the formulas: Limit of blank (LOB) = mean blank +1.645 × standard deviation (SD) of the blank; LOD = LOB +1.645 × SD of the lowest concentration sample that showed a significant difference compared with the control (Liu et al., 2024; Ma et al., 2024; Guo et al., 2025b). The calculated LOD is marked with an asterisk in the relevant dose–response curves. All statistical analyses were performed using SPSS software version 24.0 (SPSS Inc., Chicago, IL, United States).

For all biosensing assays, three independent biological replicates (n = 3) were evaluated for each construct at each concentration and time point. Time-response assays included measurements at eight time points (0–7 h), dose-response curves comprised 12–15 Pb(II) concentrations (0–800 µM), and selectivity assays tested four metal ions at eight concentrations each.

## Results and discussion

### Experimental design rationale

**FIGURE 1:**
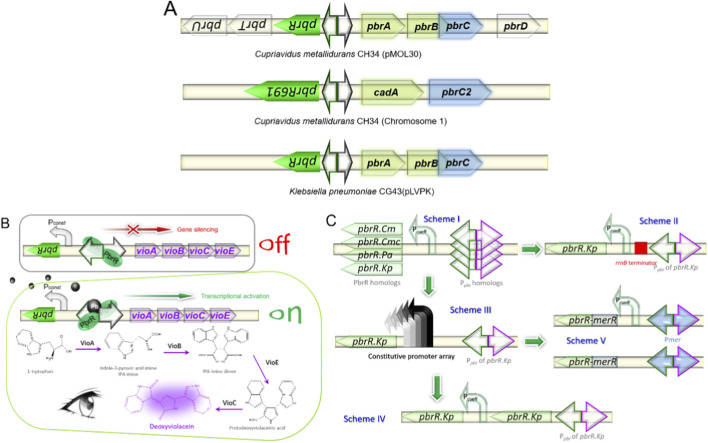
Screening and optimizing *pbr* operon-derived pigment-based biosensors in diverse bacterial species. **(A)** Schematic illustration of *pbr* operons from various bacterial species used as genetic templates. The divergent *pbr* promoter is indicated by a double-headed arrow, the regulator gene is colored green, and the structural genes are depicted in different colors. **(B)** A mechanistic overview of the whole-cell biosensor’s response to Pb(II), leading to the production of purple color signals. The dimeric PbrR protein binds to the divergent *pbr* promoter, thereby inhibiting the transcription of the *vioABCE* gene cluster. Upon Pb(II) binding to the dimeric PbrR, it transitions from a repressor to an activator, initiating the transcription of the *vioABCE* gene cluster. The resulting VioA, VioB, VioE, and VioC enzymes then catalyze the biosynthesis of DV from the substrate L-tryptophan. **(C)** Detailed assembly strategies for the biosensing constructs are outlined as follows: Scheme I employs a screening method based on a decoupled genetic circuit. Scheme II: Incorporation of a transcription terminator to reduce background noise. Scheme III: Utilization of a series of constitutive promoters to regulate the expression levels of PbrR.Kp. Scheme IV: Modulation of PbrR.Kp levels are determined through dual coding gene copies. Scheme V: Integrating a hybrid operon system combining *pbr* and *mer* operons.

In our search for excellent sensory elements, we investigated the genetic diversity of environmental bacteria that have adapted to stress from heavy metals through genetic transfer mechanisms (Jung and Lee, 2019; Grosse et al., 2023). While many such bacteria have been genetically annotated, a significant subset still requires characterization (Hui et al., 2023). Our focus was on the *pbr* operons, which orchestrate lead detoxification pathways. As depicted in Figure 1A, a spectrum of Pb resistance genes is orchestrated by the PbrR regulator within the *pbr* operons. Prominent among these is the intricate *pbrUTRABCD* operon, which is resident on the pMOL30 plasmid of *Cupriavidus metallidurans* CH34 and has been extensively studied (Borremans et al., 2001). Furthermore, two PbrR homologs, designated PbrR691 and PbrR710, were discerned across the chromosomes of this bacterium, with PbrR691 being part of a contiguous *pbr* operon (Taghavi et al., 2009). The crystallographic structure of PbrR691 has been elucidated, providing insights into its metalloregulatory function (Huang et al., 2016).

Although biosensors leveraging PbrR homologs from pMOL30, a plasmid of *C. metallidurans* CH34, and the pLVPK plasmid of *Klebsiella pneumoniae* CG43 have been successfully implemented (Wei et al., 2014; Bereza-Malcolm et al., 2016; Guo Y. et al., 2019; Hui et al., 2020a; Hui et al., 2020b; Hui et al., 2021a; Hui C. Y. et al., 2022; Guo et al., 2024a), the biosensing potential of *pbr* operons from other sources remains largely unexplored. To address this gap, we initiated a comparative analysis of four distinct *pbr* elements (Table 2), two of which were previously uncharacterized. This analysis was conducted using a decoupled genetic circuit (Zhang et al., 2024), with the DV biosynthetic gene cluster serving as an amplified visual reporter (Hui et al., 2024b; Liu et al., 2024), as validated in prior research (Figure 1B). The selection of the optimal *pbr* element was based on a critical evaluation of biosensing metrics, including signal-to-noise ratio, specificity, and dose-responsiveness. Subsequently, we employed five distinct strategies (Figure 1C) to refine the biosensing performance, with a focus on minimizing background noise and optimizing dose responsiveness.

### Identification and characterization of four lead-responsive elements

**FIGURE 2:**
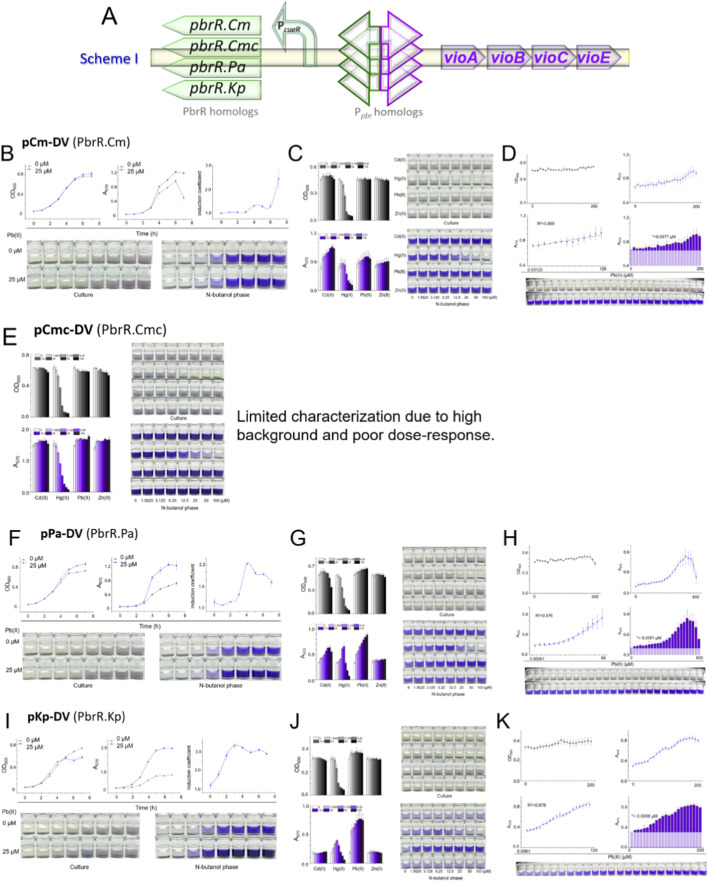
Comprehensive evaluation of four pbr operon-derived biosensors. **(A)** Scheme I schematic. **(B–D)** Characterization of TOP10/pCm-DV (PbrR.Cm): **(B)** temporal response, **(C)** selectivity, **(D)** dose-dependent behavior with LOD determination (white area = background; asterisk = LOD). **(E)** Selectivity of TOP10/pCmc-DV (PbrR.Cmc). **(F–H)** Characterization of TOP10/pPa-DV (PbrR.Pa): **(F)** temporal response, **(G)** selectivity, **(H)** dose-response. **(I–K)** Characterization of TOP10/pKp-DV (PbrR.Kp): **(I)** temporal response, **(J)** selectivity, **(K)** dose-response.

By Scheme I (as depicted in Figure 2A), we conducted an exhaustive comparison to elucidate the subtle variations in biosensing performance associated with the distinct *pbr* operons under investigation. The PbrR homologs derived from the *Cupriavidus metallidurans* CH34 plasmid pMOL30, *Cupriavidus metallidurans* CH34 chromosome 1, *Pseudomonas aeruginosa* strain PaLo1 chromosome, and *Klebsiella pneumoniae* CG43 plasmid pLVPK were designated as PbrR.Cm, PbrR.Cmc, PbrR.Pa, and PbrR.Kp, respectively. A summary of detection ranges, specificity, and LODs is provided in Supplementary Table S2. Based on superior pKp-DV performance (lowest LOD, broadest range), subsequent optimization strategies (Schemes II-V) used pKp-DV as a template, with each hybrid/optimized construct independently built and validated through fresh transformation rather than extrapolating from prior data.

The time-dependent biosensing responses of the engineered bacteria expressing different PbrR homologs. As illustrated in Figure 2B, PbrR.Cm demonstrated that both 0 μM and 25 μM Pb(II)-induced DV signals increased with an extension of the induction period, with negligible differences in bacterial proliferation observed between the groups. Consequently, an induction period of 4 h was selected for subsequent experiments, as it exhibited the highest induction coefficient within the shortest timeframe, thereby optimizing the detection process and minimizing the increase in background signal.

The selectivity assay, presented in Figure 2C, revealed that the tested metal ions, except Hg(II), did not exert significant cytotoxic effects. However, the assay also highlighted a considerable background signal in the absence of any metal exposure, with DV absorbance readings averaging around 0.5. Regrettably, PbrR.Cm exhibited poor selectivity, responding more robustly to Cd(II) than to Pb(II). Previous attempts to enhance the Pb(II) selectivity of PbrR.Cm through directed evolution and genetic circuit engineering has only achieved limited success (Jia et al., 2018; Jia et al., 2020). The dose-response relationship for PbrR.Cm was further compromised by the elevated background noise and other unidentified factors, resulting in a high LOD at 0.0977 μM, as depicted in Figure 2D. Therefore, as employed in this study, the screening strategy for Pb(II)-specific PbrR homologs emerges as a potentially efficacious approach to refine the selectivity and performance of whole-cell biosensors.

The Pb(II)-binding characteristics and crystallographic structure of PbrR691 have been extensively investigated, culminating in the development of a protein-based Pb(II) sensor that leverages PbrR691 (Chen et al., 2007; Huang et al., 2016). Despite these advances, the application of PbrR691 in constructing whole-cell biosensors has yet to be explored. This study harnessed the DV biosynthetic gene cluster as a visual reporter controlled by a PbrR.Cmc (alias for PbrR691)-containing operon. The selectivity of the engineered bacterial biosensor, TOP10/pCmc-DV, was subsequently evaluated. As depicted in Figure 2E, bacterial growth showed a decreasing trend with increasing concentrations of metal ions. However, pronounced DV leakage was detected, with absorbance measurements peaking at approximately 1.4. While Pb(II) and Zn(II) stimulation increased DV production, the dose-response dynamics were not distinctly discernible.

Contrasting with other *pbr* operons, the intergenic region housing the divergent *pbr* promoter in PbrR.Cmc is notably truncated (refer to Supplementary Figure S1 in Supplementary Material), which may account for the diminished performance of PbrR.Cmc-based biosensors. The absence of critical regulatory sequences could undermine the biosensor’s efficacy. Consequently, through database mining, we identified PbrR.Pa shares significant homology with PbrR.Cmc, but is distinguished by a more extended *pbr* promoter. This characteristic suggested the presence of additional regulatory elements that could enhance whole-cell biosensing performance. A novel bacterial biosensor, TOP10/pPa-DV, was consequently developed, and its functional attributes were thoroughly characterized in subsequent analyses.

As anticipated, the temporal biosensing profile of the TOP10/pPa-DV strain (Figure 2F) demonstrated marked enhancement relative to the TOP10/pCmc-DV counterpart. A notably amplified 25 μM Pb(II)-induced DV signal was observed at the 4-h mark, surpassing that of the 0 μM Pb(II)-induced signal, thus warranting its selection for subsequent investigations. The selectivity assay outcomes for TOP10/pPa-DV are presented in Figure 2G. The background DV absorbance at 570 nm was effectively reduced to 0.35, indicating a lower baseline signal compared to the levels observed in Figures 2C,E. Notably, the biosensor TOP10/pPa-DV exhibited a robust and selective dose response to Pb(II) and Cd(II), with a hierarchy of responsiveness from high to low.

Moreover, a pronounced elevation in the DV signal was detected in response to elevated Hg(II) concentrations, precisely at 6.25 and 12.5 μM, despite the concomitant observation of significant bacterial growth inhibition under such conditions. With the background noise curtailed, an enhanced dose-response relationship was discernible in Figure 2H. Utilizing nonlinear regression analysis, a broad quantitative detection range spanning from 0.00381 to 50 μM was established, with the LOD being commendably reduced to 0.0061 μM. These improvements underscore the potential of PbrR.Pa-based biosensors provide a sensitive and wide-ranging detection platform for heavy metal ions, mainly Pb(II).

In our prior work, we successfully engineered a DV-based bacterial biosensor utilizing PbrR.Kp within a naturally coupled circuit, achieving a LOD for Pb(II) of 0.00293 μM and establishing a robust linear dose-response model across a broad range (0.00293–6 μM) (Hui C. Y. et al., 2022). Building upon these findings, we have constructed the TOP10/pKp-DV biosensor, leveraging PbrR.Kp within a distinct decoupled circuit. This novel configuration has yielded a markedly enhanced time-response profile (Figure 2I) compared to the three biosensors previously discussed. Notably, a discernible difference in the DV signal between 0 and 25 μM Pb(II) induction was observed as early as 2 h, with the induction coefficient peaking at 3 h, thus informing the selection of this time point for subsequent analyses.

Contrary to expectations, the background DV signal in the TOP10/pKp-DV biosensor escalated to approximately 0.3 (Figure 2J), surpassing the background levels of approximately 0.1 observed in our earlier biosensor that employed a natural coupled circuit (Hui C. Y. et al., 2022). At the same time, the previous study (Hui C. Y. et al., 2022) did not extensively explore the selectivity of PbrR.Kp, our current study reveals that the TOP10/pKp-DV biosensor exhibits a high response to Pb(II) but also shows a notable response to Hg(II) (Figure 2J). Despite this, the biosensor TOP10/pKp-DV exhibited an impressive dose response to Pb(II) across an extended range from 0.0061 to 50 μM (Figure 2K), significantly broadening the dynamic detection range compared to our previously reported construct. Moreover, the LOD was commendably reduced to 0.0008 μM. These results suggest that the decoupled circuit design enhances the biosensor’s sensitivity and expands its Pb(II) response concentration range, albeit at the cost of increased background noise.

Although the three cysteine ligands required for Pb(II) coordination (C79, C114, C123 in PbrR.Cm numbering) are strictly conserved in PbrR.Kp residues flanking this core, which cluster around the metal-binding pocket, differ subtly from those in the other homologs (Hui et al., 2023). These local variations may slightly constrict the cavity, favouring the distorted trigonal Pb(II)–thiolate geometry while disfavouring the bulkier Cd(II)/Zn(II) tetrahedral complexes (Hobman et al., 2012). This structural fine-tuning explains the higher Pb(II) selectivity and the lowest LOD observed with the pLVPK-derived system.

Addressing the challenges of background noise reduction, sensitivity enhancement, and expanding the Pb(II) detection range will be pivotal for advancing our biosensing technology. In the subsequent phase of our study, we intend to tackle these issues through innovative genetic circuit modifications based on the pKp-DV construct.

### Transcriptional terminator-mediated mitigation of background noise

**FIGURE 3:**
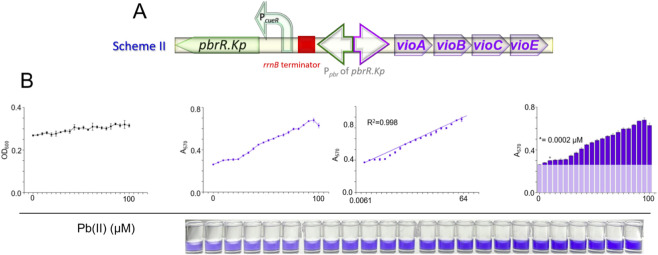
Biosensing performance of engineered bacteria modified with Scheme II. **(A)** Schematic depiction of Scheme II, detailing the strategic integration of a transcription terminator to suppress basal expression levels and thereby reduce background noise. **(B)** Dose-response characteristics of the refined biosensor strain TOP10/pTer-Kp-DV. The asterisk represents the calculated detection limit.

The strategic placement of a transcription terminator is a well-established approach to attenuate basal transcriptional noise (Wang and Buck, 2012; Du et al., 2019). In this context, we introduced a potent transcription terminator (*rrnB* terminator) upstream of the divergent *pbr* promoter within the pKp-DV construct using overlap PCR, resulting in the generation of the pTer-Kp-DV biosensor (Figure 3A). The specific sequence of the terminator and its insertion site are detailed in Supplementary Table S1. This modified construct was then integrated into the same host strain, yielding the bacterial biosensor TOP10/pTer-Kp-DV. The subsequent dose-response analysis is depicted in Figure 3B.

Consistent with our hypothesis, introducing the transcription terminator resulted in a modest reduction in the background DV signal, thereby affording a marginal enhancement in sensor sensitivity. This refinement resulted in a lower LOD, achieving a value of 0.0002 μM. However, the dynamic range for quantitative Pb(II) detection remained comparable to that of the TOP10/pKp-DV biosensor.

This finding underscores the potential of transcription terminators in fine-tuning the signal-to-noise ratio of whole-cell biosensors. Despite the modest improvement, these results underscore the need for additional optimization strategies to achieve a more pronounced reduction in background noise and to extend the detection range.

### Regulator dosage and its impact on biosensing performance

The concentration of metalloregulatory proteins has been shown to modulate the sensitivity of MerR-based bacterial biosensors and reduce background noise in ArsR-based systems (Guo M. et al., 2019; Liu et al., 2024). As a member of the MerR family (Hui et al., 2023), the Pb(II)-responsive metalloregulator PbrR’s influence on the performance of PbrR-based bacterial biosensors was investigated. The hypothesis was that adjusting the expression levels of PbrR could enhance biosensor characteristics.

The strength of a constitutive promoter can be altered by modifying the RNA polymerase binding site (Brewster et al., 2012). Based on this finding, a series of constitutive promoters has been created. The number in the promoter name can determine the promoter’s strength. Therefore, the constitutive promoters used in this study, listed from weakest to strongest, are: 302, 406, 479, 535, 637, 699, and J23119.

**FIGURE 4:**
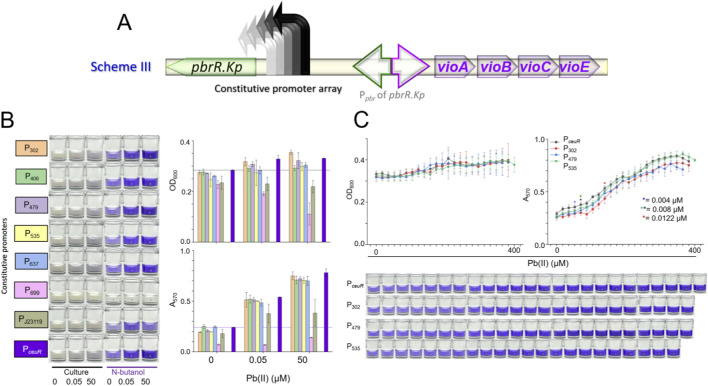
Biosensing performance of engineered bacteria using Scheme III. **(A)** Schematic of constructs in which PbrR is driven by graded constitutive promoters. **(B)** Representative photographs (left) and quantitative data (right) of bacterial cultures/n-butanol extracts after 3 h induction with 0, 0.05, or 50 μM Pb(II); dashed lines denote baseline levels of the parental TOP10/pKp-DV strain. **(C)** Dose–response curves for the indicated biosensors; LODs are marked with asterisks.

To test this hypothesis, a series of constructs were generated using a decoupled circuit, where a range of constitutive promoters was employed to vary the expression levels of the PbrR regulator (Figure 4A). The strength of these promoters was estimated based on their designations; however, it is acknowledged that promoter strength can fluctuate with different target genes (Brewster et al., 2012). Additionally, the widely used strong promoter PJ23119, commonly employed in synthetic biology applications (Liu et al., 2024), was included to assess the impact of PbrR overexpression.

As depicted in Figure 4B, the results revealed variable yet statistically insignificant reductions in the background DV signal among the biosensor constructs. Notably, the biosensor TOP/p699-Kp-DV failed to produce DV under various Pb(II) concentrations, potentially due to mRNA structural interference that silences gene expression (Sato et al., 2023). Furthermore, the biosensor TOP/pJ23119-Kp-DV exhibited a weak DV signal in response to Pb(II), possibly because the overexpression of PbrR depletes cellular resources, thereby diminishing pigment synthesis. This observation is consistent with previous studies (Liu et al., 2024; Guo et al., 2025b) showing that while strong promoters can reduce background noise by driving high expression of metalloregulators, they often also lead to a decrease in biosensing signals, potentially due to the depletion of cellular resources associated with overexpression.

A comprehensive evaluation of the dose-response curves for biosensors with different PbrR expression levels is presented in Figure 4C. Slight variations in both bacterial growth and dose-response profiles were observed across the four biosensors. The minor changes in detection limits are likely due to experimental variability rather than significant biological effects. Given that the introduction of strong promoters led to a reduction in pigment signals, we selected four weaker promoters (P_302_, P_479_, P_535_, and P_*ceuR*_) to systematically investigate the overall dose-response relationships and avoid the negative impact of strong promoter-driven overexpression on biosensor performance.

While the expression level of the metalloregulator PbrR did not significantly influence the biosensing performance in this study, these findings provide valuable insights into the complex interplay between regulator dosage, cellular metabolism, and biosensor output. These observations guide future efforts in optimizing biosensor design by highlighting the need for a balanced approach to regulator expression levels.

Previous studies have demonstrated that tuning the expression level of Hg(II)-responsive MerR via a graded series of constitutive promoters yields a parabolic relationship between MerR abundance and the slope of the calibration curve, thereby enabling customizable sensitivity (Guo M. et al., 2019; Guo et al., 2025c). Similarly, increasing the dosage of As(III)-responsive ArsR—an ArsR/SmtB family repressor—was shown to proportionally reduce the background signal of pigment-based arsenic biosensors, owing to tighter occupancy of the ArsR binding site (Liu et al., 2024; Guo et al., 2025b). These precedents prompted us to test whether the same constitutive promoter-array approach could attenuate the background noise of our Pb(II) sensor.

However, none of the PbrR expression levels examined here significantly reduced DV leakage (Figure 4B). This discrepancy can be rationalized by considering the distinct modes of action of the three regulator families. ArsR functions as a classic derepressor: low basal expression often results in incomplete occupation of the operator, and simply elevating ArsR levels is sufficient to repress background transcription (Hui et al., 2024a). However, MerR and its homolog PbrR are dual-function activators/repressors bound to their divergent promoters, irrespective of metal presence (Hui et al., 2024b; Guo and Hui, 2025). In the absence of Pb(II), the PbrR dimer maintains the promoter in a repressed yet “poised” state; Pb(II) binding induces a conformational change that converts PbrR into an activator, recruiting RNA polymerase and initiating transcription (Hui et al., 2023). Consequently, the persistent low-level expression of the downstream operon may not arise from insufficient PbrR abundance, but rather from intrinsic promoter leakiness that is refractory to further repressor saturation. Such basal expression could be evolutionarily advantageous, allowing cells to stand against sudden lead exposure.

Although the constitutive-promoter array successfully modified the MerR and ArsR-based biosensors in the studies above, we acknowledge that direct quantification of *pbrR* transcripts was not performed in this study. Future work will therefore include qRT-PCR validation of PbrR mRNA levels across the promoter series, providing a quantitative framework to guide subsequent circuit optimization.

### Impact of hybrid genetic circuits on biosensing performance

Previous studies have reported a negligible background pigment signal in PbrR-based bacterial biosensors utilizing natural coupled circuits (Hui et al., 2020a; Hui et al., 2021a; Hui C. Y. et al., 2022). In contrast, this study has successfully expanded the quantitative detection range for Pb(II) using a decoupled circuit. This observation led to the hypothesis that a hybrid genetic circuit, combining features of both coupled and decoupled designs, might offer a synergistic advantage by reducing background signals and broadening the detection range.

**FIGURE 5:**
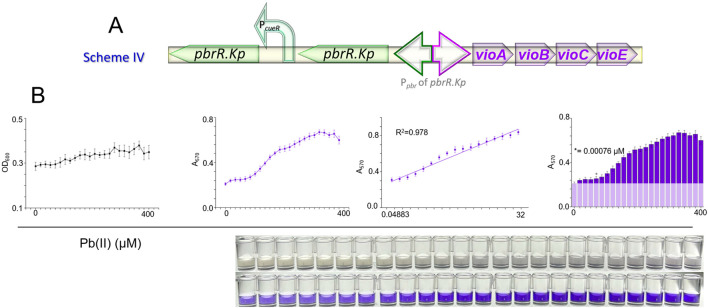
Biosensing performance of engineered bacteria utilizing scheme IV. **(A)** Schematic overview of Scheme IV, demonstrating the approach to modulate the levels of the PbrR.Kp transcription factor by introducing dual copies of the coding gene. **(B)** Dose-response analysis of the biosensor strain TOP10/pKp-2P-DV.

To test this hypothesis, we constructed Scheme IV, as illustrated in Figure 5A. This Scheme aimed to integrate the minimal background noise characteristic of the coupled circuit with the expanded detection range afforded by the decoupled circuit. However, the results were less promising than they were. The combination of the two genetic circuits did not yield significant improvements in background noise, detection range, or LOD, as evidenced in Figure 5B.

This outcome suggests that the interaction between coupled and decoupled circuits is complex and may not automatically result in a composite benefit. The lack of significant improvement could be attributed to several factors, including potential regulatory conflicts or inhibitory effects when both circuit types are present simultaneously within the same biosensor.

While merging coupled and decoupled circuits was a rational approach based on individual circuit performances, the actual application needed to meet the expected outcomes. These results underscore the need for a nuanced understanding of genetic circuit interactions and the importance of empirical validation in synthetic biology.

### Lack of enhancement in biosensing performance with heterologous operon integration

While prior research has established that chimeric MerR-family regulators can enhance biosensing performance by improving sensitivity and selectivity (Ghataora et al., 2023), our attempt to integrate heterologous operons did not yield anticipated improvements. MerR-family regulators possess a DNA-binding domain (DBD) and a metal-binding domain (MBD), which are crucial for metal-binding specificity (Hui et al., 2024b). It has been demonstrated that displaying a truncated version of PbrR, specifically its MBD, can enhance Pb(II) capture capacity (Hui C. et al., 2018; Hui C. Y. et al., 2018). The Hg(II)-responsive regulator MerR, renowned for its high specificity and minimal basal transcription (Guo et al., 2021; Zhang et al., 2021; Hui C. et al., 2022), presents an opportunity to create a regulator with combined attributes.

**FIGURE 6:**
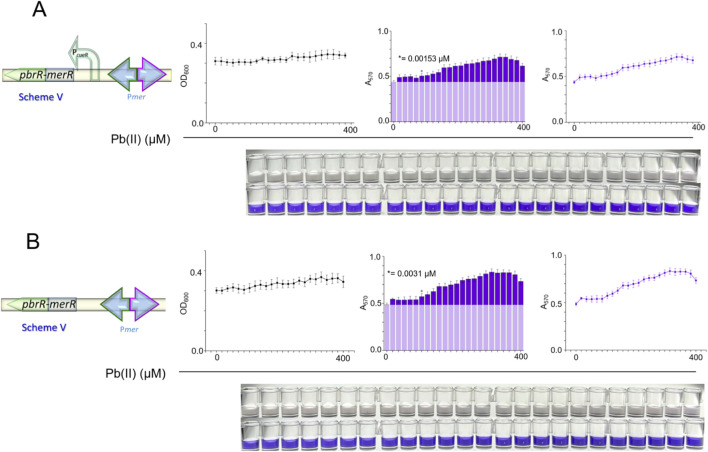
Biosensing performance through the integration of heterologous operons. Schematic representations of the hybrid operon systems that combine elements from both *pbr* and *mer* operons. Dose-response relationship for the biosensor TOP10/pCON-MP-DV using decoupled circuits **(A)** and TOP10/pNAT-MP-DV using naturally coupled circuits **(B)**.

To merge the Pb(II)-binding specificity of PbrR with the tight regulation of MerR, we engineered a chimeric regulator, PbrRMerR, by fusing the MBD of PbrR with the DBD of MerR. The sequence of the chimeric protein PbrRMerR is provided in Supplementary Figure S2 in the Supplementary Material, clearly marking the MBD and DBD regions. Utilizing the DBD of MerR, we employed the divergent *mer* promoter and constructed biosensing constructs using decoupled (Figure 6A) and natural coupled (Figure 6B) genetic circuits. Regrettably, these constructs did not exhibit a reduced background DV signal or improved dose-response relationships in either circuit.

The chimeric PbrRMerR construct described in Figure 6 represents our first attempt to exploit the near-zero background of the native *mer* system (Hui et al., 2024b). Combining the MerR DNA-binding domain with the Pb(II)-binding domain of PbrR with the divergent *mer* promoter did yield a marginal Pb(II) response, yet the background A_570_ rose from ∼0.3 (pKp-DV) to ∼0.5 (pCON-MP-DV). According to our prior experience, this modest increase is sufficient to blunt the slope of the calibration curve and obscure the LOD (Liu et al., 2024). The underlying reasons likely stem from the evolutionary fine-tuning of each natural operon. The regulatory mechanisms that keep the *mer* promoter inactive may be disrupted when foreign effector-binding sites are added. Additionally, some subtle transcriptional interference between the *pbr* and *mer* elements may not be dismissed.

In summary, the reliability of our comparative evaluation was established through multi-level validation. All biosensing assays were performed in triplicate with independently transformed *E. coli* TOP10 colonies. Statistical analysis confirmed significance (*P* < 0.05) for all performance differences. The four *pbr* operons were simultaneously tested under identical conditions, eliminating batch effects. The pKp-DV’s superiority was confirmed across three independent experimental repeats. Each genetic modification was independently constructed and fully re-characterized rather than extrapolating from prior data. The best-performing pKp-DV biosensor achieved a 4-fold lower LOD and 8-fold wider dynamic range compared to previously reported *pbr*-based DV biosensors, confirming genuine performance gains rather than experimental artifacts.

Future efforts should therefore explore higher-threshold pigments such as indigoidine or indigo, which our recent studies have shown to generate markedly lower background signals (Guo et al., 2024a; Guo et al., 2025b), and implement logic-gated transcriptional circuits (Liu et al., 2022) to insulate the sensor from unintended crosstalk.

While the current study establishes a robust proof-of-concept for Pb(II) detection with our *pbr*-operon-based DV biosensor, its readiness for field deployment still hinges on systematic validation under environmental stresses such as fluctuating pH, temperature, and complex water matrices. Encouragingly, our recent pigment-based sensor for organophosphate pesticides (Wu et al., 2024) has already demonstrated prolonged cold-storage stability and reliable performance across freshwater, seawater, and soil leachates, providing a transferable framework for the forthcoming optimization of this lead biosensor.

## Conclusion

The present study successfully engineered whole-cell biosensors for Pb(II) detection using diverse *pbr* operons, with the *Klebsiella pneumoniae* CG43 plasmid pLVPK-derived operon exhibiting the highest sensitivity and specificity. Genetic circuit engineering modestly improved sensor performance by reducing background noise. The developed biosensors offer a promising approach for rapid and cost-effective Pb(II) monitoring in environmental settings, highlighting the potential of synthetic biology in environmental sensing applications. Despite these advances, several limitations warrant acknowledgment. First, the study was conducted exclusively in laboratory-adapted *E. coli* TOP10 under controlled conditions using LB medium. Performance in real-world environmental matrices (e.g., seawater, soil leachates) remains to be validated. Second, while the pKp-DV construct showed the highest sensitivity, its elevated background signal in decoupled circuits represents a trade-off that may limit the detection of trace Pb(II) in turbid samples. Third, the pigment-based readout exhibits slower response kinetics (3–4 h) compared to electrochemical biosensors. Fourth, genetic circuit modifications did not achieve synergistic effects in hybrid designs, suggesting more sophisticated insulation mechanisms may be necessary. Future work will systematically test genetic stability under varied pH, temperature, and environmental stresses, then combine chromosomal integration with low-background pigments for robust field deployment.

## Data Availability

The raw data supporting the conclusions of this article will be made available by the authors, without undue reservation.
