# Supplementary material for: Systematic evaluation of pigment-based whole-cell lead biosensors: challenges in genetic circuit engineering and critical considerations for background noise control
Source: Front Bioeng Biotechnol. 2026 Jan 7;13:1744651. doi: 10.3389/fbioe.2025.1744651 (PMC12819690; doi:10.3389/fbioe.2025.1744651)
Supplement: Supplementary file 1 [file Table1.docx]

**Supplementary materials**

**Table. S1** DNA sequence of artificially designed Pb(II)-responsive elements used in this study.

| **Vectors**  **(inserted sites)** | **Pb(II)-responsive elements** | **Origin** |
| --- | --- | --- |
| pCm-DV  (*Bgl*II/*Xba*I) | AGATCTCTAGTCGCTTGGATGGGCGGTGGTCCCCCGCGTATCACACACGCAGTCCGACAGTCCCTGCAGAATCCCGCACGATTGGGCGGGCCTGGCACCAGAACAGGCTTCGCGCAGTTCCACCAAATGGTGCTTCAGTTCGAGCAAAGCTCCGATCCGAGATTCGACCTGACGGATGTGCTCATCCAAGAGCATATTGACTTCACCGCAATCCTGGTCGGGCCGCTTCCGGTAACTCAATAAGGTCCGTACGTCGCTCAACGGCATATCCAGAGACCGGCAGTGACGAATGAACTGCAAGCGCTCCACGTGCTCCTCGCCATACAGGCGAAAATTCCCCCGGCTGCGGCCCGGCGGCGGCAACAGCCCTTCTTGTTCGTAGAAGCGAATGGTCACCACCGGGCATGCGGTGCGCTTGGCAAGCTCGCCGATCTGGATATTCATCGTTCGCGCAACGCCTCCTGGTACCTGTGCTTTGTTAAAGGGTTTCAACAGATATTCAAGGCGTCGGATGGGAGATGTCTTGACTCTATAGTAACTAGAGGGTGTTAAATCGGCAACGCGAGATGAATACACACAAGGGGTTGCCTCTAGA | *Cupriavidus metallidurans* CH34 plasmid pMOL30 |
| pCmc-DV  (*Bgl*II/*Xba*I) | AGATCTTCAGGCGGGCTCGGCCAAGCTGTTGAGAATGCCGCACTCGCGCGAGGTTCGGGCGCTATCGCAGGAGCGTCGCAGATCCATTAACTCGCGCTCCAAGGCGCGCAATTCCTTCATCTTGGTCCGCACTTGCGCGATATGAGCGTCGACCAGCGCGTTCACCTCGCCGCAGCCCAACTCTGGCCGATCCCGTAAGTTCAGCAGTTGACGGATCTCATCCAGCGTCATGTCCTTCGCCCGGCAGCGGCGGATGAACAGCAAGCGCTGCAAATGGACTTCGTCATAGAGCCTGAAGTTGCCCTCGCTACGTGCAGGCTCGGGCAGCAAGCCTTCTGACTCGTAAAAGCGCACGGTCTGCACCAAGCAATCTGCCTTCTTGCCCAGTTCACCGATCCGCATCATCGTTCGCGCAACGCCTCCTGGTACCTGTGCTTTGTTAAAGGGTTTCAACAGATATTCAAGGTTGCTTCCTATAAAAAACTTGACTCTATATCTACTAGAGGTTTTCTATCTAGA | *Cupriavidus metallidurans* CH34 chromosome 1 |
| pPa-DV (*Bgl*II/*Xba*I) | AGATCTTCAGGCGGGCTCGGCCAAGCTGTTGAGAATGCCGCACTCGCGCGAGGTTCGGGCGCTATCGCAGGAGCGTCGCAGATCCATTAACTCGCGCTCCAAGGCGCGCAATTCCTTCATCTTGGTCCGCACTTGCGCGATATGAGCGTCGACCAGCGCGTTCACCTCGCCGCAGCCCAACTCTGGCCGATCCCGTAAGTTCAGCAGTTGACGGATCTCATCCAGCGTCATGTCCTTCGCCCGGCAGCGGCGGATGAACAGCAAGCGCTGCAAATGGACTTCGTCATAGAGCCTGAAGTTGCCCTCGCTACGTGCAGGCTCGGGCAGCAAGCCTTCTGACTCGTAAAAGCGCACGGTCTGCACCAAGCAATCTGCCTTCTTGCCCAGTTCACCGATCCGCATCATCGTTCGCGCAACGCCTCCTGGTACCTGTGCTTTGTTAAAGGGTTTCAACAGATATTCAAGGTTGCTTCCTATAAAAAACTTGACTCTATATCTACTAGAGGTTTTCTAATGATGGCATCCGGGGAAAACCTTGTCAATGAAGAGCGATCTTCTAGA | *Pseudomonas aeruginosa strain* PaLo1 chromosome |
| pKp-DV (*Bgl*II/*Xba*I) | AGATCTTTACCCAGATGTTTGACTGTTCGTGGCACTTTCACCATGGCAATTGCCCAACCCTTGCAAAATGCCGCACGCCTCTACAGATCGAGAGCCAGAACACTTCTCGCGCAAATCAACCAAGTGCCGTTTTAACTGCAACAGCGCGGACACACGCATTTCCACCTGTTGAATATGGGCCTCCAGCAGCGTGATGACCTCCCCACAGTCCTGCATCGGGTTGTCTCGCAGACCCAGCAATGCGCGAATCTCGCTCAACGTCATGTCGAGCGAACGGCAATGACGGATGAATTGCAAGCGCTCAATGTGCGCCTCACCGTACAACCGAAAGTTGCCACCGCTTCGCGCTGGCTTTGGCAGTAGCCCTTCCTTCTCGTAGTAGCGGATGGTCACGACCTCGCACCCAGAGCGCTTGGCGAGGTCGCCAATTCTGATTTCCATCGTTCGCGCAACGCCTCCTGGTACCTGTGCTTTGTTAAAGGGTTTCAACAGATATTCAAGCATCAATCTCCAATTATCACTTGACTCTATAGTGACTATAGAGATTTTAATGGAGGCTGAATAGAAGATTTTCAGGAGTTACTCTCTAGA | *Klebsiella pneumoniae* CG43 plasmid pLVPK |
| p302-Kp-DV (*Bgl*II/*Xba*I) | AGATCTTTACCCAGATGTTTGACTGTTCGTGGCACTTTCACCATGGCAATTGCCCAACCCTTGCAAAATGCCGCACGCCTCTACAGATCGAGAGCCAGAACACTTCTCGCGCAAATCAACCAAGTGCCGTTTTAACTGCAACAGCGCGGACACACGCATTTCCACCTGTTGAATATGGGCCTCCAGCAGCGTGATGACCTCCCCACAGTCCTGCATCGGGTTGTCTCGCAGACCCAGCAATGCGCGAATCTCGCTCAACGTCATGTCGAGCGAACGGCAATGACGGATGAATTGCAAGCGCTCAATGTGCGCCTCACCGTACAACCGAAAGTTGCCACCGCTTCGCGCTGGCTTTGGCAGTAGCCCTTCCTTCTCGTAGTAGCGGATGGTCACGACCTCGCACCCAGAGCGCTTGGCGAGGTCGCCAATTCTGATTTCCATtttttttacctccttaaCCACACAACATACGAGCCGGAAGCATAAAGTCTAAGGCCTGGCATCAATCTCCAATTATCACTTGACTCTATAGTGACTATAGAGATTTTAATGGAGGCTGAATAGAAGATTTTCAGGAGTTACTCTCTAGA | *Klebsiella pneumoniae* CG43 plasmid pLVPK |
| p406-Kp-DV (*Bgl*II/*Xba*I) | AGATCTTTACCCAGATGTTTGACTGTTCGTGGCACTTTCACCATGGCAATTGCCCAACCCTTGCAAAATGCCGCACGCCTCTACAGATCGAGAGCCAGAACACTTCTCGCGCAAATCAACCAAGTGCCGTTTTAACTGCAACAGCGCGGACACACGCATTTCCACCTGTTGAATATGGGCCTCCAGCAGCGTGATGACCTCCCCACAGTCCTGCATCGGGTTGTCTCGCAGACCCAGCAATGCGCGAATCTCGCTCAACGTCATGTCGAGCGAACGGCAATGACGGATGAATTGCAAGCGCTCAATGTGCGCCTCACCGTACAACCGAAAGTTGCCACCGCTTCGCGCTGGCTTTGGCAGTAGCCCTTCCTTCTCGTAGTAGCGGATGGTCACGACCTCGCACCCAGAGCGCTTGGCGAGGTCGCCAATTCTGATTTCCATtttttttacctccttaaCCACACGTTATCCGAGCCGGAAGCATAAAGTGTAAACTCGAGCATCAATCTCCAATTATCACTTGACTCTATAGTGACTATAGAGATTTTAATGGAGGCTGAATAGAAGATTTTCAGGAGTTACTCTCTAGA | *Klebsiella pneumoniae* CG43 plasmid pLVPK |
| p479-Kp-DV (*Bgl*II/*Xba*I) | AGATCTTTACCCAGATGTTTGACTGTTCGTGGCACTTTCACCATGGCAATTGCCCAACCCTTGCAAAATGCCGCACGCCTCTACAGATCGAGAGCCAGAACACTTCTCGCGCAAATCAACCAAGTGCCGTTTTAACTGCAACAGCGCGGACACACGCATTTCCACCTGTTGAATATGGGCCTCCAGCAGCGTGATGACCTCCCCACAGTCCTGCATCGGGTTGTCTCGCAGACCCAGCAATGCGCGAATCTCGCTCAACGTCATGTCGAGCGAACGGCAATGACGGATGAATTGCAAGCGCTCAATGTGCGCCTCACCGTACAACCGAAAGTTGCCACCGCTTCGCGCTGGCTTTGGCAGTAGCCCTTCCTTCTCGTAGTAGCGGATGGTCACGACCTCGCACCCAGAGCGCTTGGCGAGGTCGCCAATTCTGATTTCCATtttttttacctccttaaCCACACATTATACGAGCCGGAAGCATAAAGTCTTAACTCGAGCATCAATCTCCAATTATCACTTGACTCTATAGTGACTATAGAGATTTTAATGGAGGCTGAATAGAAGATTTTCAGGAGTTACTCTCTAGA | *Klebsiella pneumoniae* CG43 plasmid pLVPK |
| p535-Kp-DV (*Bgl*II/*Xba*I) | AGATCTTTACCCAGATGTTTGACTGTTCGTGGCACTTTCACCATGGCAATTGCCCAACCCTTGCAAAATGCCGCACGCCTCTACAGATCGAGAGCCAGAACACTTCTCGCGCAAATCAACCAAGTGCCGTTTTAACTGCAACAGCGCGGACACACGCATTTCCACCTGTTGAATATGGGCCTCCAGCAGCGTGATGACCTCCCCACAGTCCTGCATCGGGTTGTCTCGCAGACCCAGCAATGCGCGAATCTCGCTCAACGTCATGTCGAGCGAACGGCAATGACGGATGAATTGCAAGCGCTCAATGTGCGCCTCACCGTACAACCGAAAGTTGCCACCGCTTCGCGCTGGCTTTGGCAGTAGCCCTTCCTTCTCGTAGTAGCGGATGGTCACGACCTCGCACCCAGAGCGCTTGGCGAGGTCGCCAATTCTGATTTCCATtttttttacctccttaaCCACACAACATACGAGCCGGAAGCATAAAGTGTAAAGCCTGGCATCAATCTCCAATTATCACTTGACTCTATAGTGACTATAGAGATTTTAATGGAGGCTGAATAGAAGATTTTCAGGAGTTACTCTCTAGA | *Klebsiella pneumoniae* CG43 plasmid pLVPK |
| p637-Kp-DV (*Bgl*II/*Xba*I) | AGATCTTTACCCAGATGTTTGACTGTTCGTGGCACTTTCACCATGGCAATTGCCCAACCCTTGCAAAATGCCGCACGCCTCTACAGATCGAGAGCCAGAACACTTCTCGCGCAAATCAACCAAGTGCCGTTTTAACTGCAACAGCGCGGACACACGCATTTCCACCTGTTGAATATGGGCCTCCAGCAGCGTGATGACCTCCCCACAGTCCTGCATCGGGTTGTCTCGCAGACCCAGCAATGCGCGAATCTCGCTCAACGTCATGTCGAGCGAACGGCAATGACGGATGAATTGCAAGCGCTCAATGTGCGCCTCACCGTACAACCGAAAGTTGCCACCGCTTCGCGCTGGCTTTGGCAGTAGCCCTTCCTTCTCGTAGTAGCGGATGGTCACGACCTCGCACCCAGAGCGCTTGGCGAGGTCGCCAATTCTGATTTCCATtttttttacctccttaaCCACACATTATACGAGCCGGAAGCATAAAGTGTAATCTCGAGCATCAATCTCCAATTATCACTTGACTCTATAGTGACTATAGAGATTTTAATGGAGGCTGAATAGAAGATTTTCAGGAGTTACTCTCTAGA | *Klebsiella pneumoniae* CG43 plasmid pLVPK |
| p699-Kp-DV (*Bgl*II/*Xba*I) | AGATCTTTACCCAGATGTTTGACTGTTCGTGGCACTTTCACCATGGCAATTGCCCAACCCTTGCAAAATGCCGCACGCCTCTACAGATCGAGAGCCAGAACACTTCTCGCGCAAATCAACCAAGTGCCGTTTTAACTGCAACAGCGCGGACACACGCATTTCCACCTGTTGAATATGGGCCTCCAGCAGCGTGATGACCTCCCCACAGTCCTGCATCGGGTTGTCTCGCAGACCCAGCAATGCGCGAATCTCGCTCAACGTCATGTCGAGCGAACGGCAATGACGGATGAATTGCAAGCGCTCAATGTGCGCCTCACCGTACAACCGAAAGTTGCCACCGCTTCGCGCTGGCTTTGGCAGTAGCCCTTCCTTCTCGTAGTAGCGGATGGTCACGACCTCGCACCCAGAGCGCTTGGCGAGGTCGCCAATTCTGATTTCCATtttttttacctccttaaCCACACATTATACGAGCCGGAAGCATAAAGTGTAAACTCGAGCATCAATCTCCAATTATCACTTGACTCTATAGTGACTATAGAGATTTTAATGGAGGCTGAATAGAAGATTTTCAGGAGTTACTCTCTAGA | *Klebsiella pneumoniae* CG43 plasmid pLVPK |
| pJ23119-Kp-DV (*Bgl*II/*Xba*I) | AGATCTTTACCCAGATGTTTGACTGTTCGTGGCACTTTCACCATGGCAATTGCCCAACCCTTGCAAAATGCCGCACGCCTCTACAGATCGAGAGCCAGAACACTTCTCGCGCAAATCAACCAAGTGCCGTTTTAACTGCAACAGCGCGGACACACGCATTTCCACCTGTTGAATATGGGCCTCCAGCAGCGTGATGACCTCCCCACAGTCCTGCATCGGGTTGTCTCGCAGACCCAGCAATGCGCGAATCTCGCTCAACGTCATGTCGAGCGAACGGCAATGACGGATGAATTGCAAGCGCTCAATGTGCGCCTCACCGTACAACCGAAAGTTGCCACCGCTTCGCGCTGGCTTTGGCAGTAGCCCTTCCTTCTCGTAGTAGCGGATGGTCACGACCTCGCACCCAGAGCGCTTGGCGAGGTCGCCAATTCTGATTTCCATtttttttacctccttaaCCTAGCATTATACCTAGGACTGAGCTAGCTGTCAAGCATCAATCTCCAATTATCACTTGACTCTATAGTGACTATAGAGATTTTAATGGAGGCTGAATAGAAGATTTTCAGGAGTTACTCTCTAGA | *Klebsiella pneumoniae* CG43 plasmid pLVPK |
| pTer-Kp-DV (*Bgl*II/*Xba*I) | AGATCTTTACCCAGATGTTTGACTGTTCGTGGCACTTTCACCATGGCAATTGCCCAACCCTTGCAAAATGCCGCACGCCTCTACAGATCGAGAGCCAGAACACTTCTCGCGCAAATCAACCAAGTGCCGTTTTAACTGCAACAGCGCGGACACACGCATTTCCACCTGTTGAATATGGGCCTCCAGCAGCGTGATGACCTCCCCACAGTCCTGCATCGGGTTGTCTCGCAGACCCAGCAATGCGCGAATCTCGCTCAACGTCATGTCGAGCGAACGGCAATGACGGATGAATTGCAAGCGCTCAATGTGCGCCTCACCGTACAACCGAAAGTTGCCACCGCTTCGCGCTGGCTTTGGCAGTAGCCCTTCCTTCTCGTAGTAGCGGATGGTCACGACCTCGCACCCAGAGCGCTTGGCGAGGTCGCCAATTCTGATTTCCATCGTTCGCGCAACGCCTCCTGGTACCTGTGCTTTGTTAAAGGGTTTCAACAGATATTCAATACAGATTAAATCAGAACGCAGAAGCGGTCTGATAAAACAGAATTTGCCTGGCGGCAGTAGCGCGGTGGTCCCACCTGACCCCATGCCGAACTCAGAAGTGAAACGCCGTAGCGCCGATGGTAGTGTGGGGTCTCCCCATGCGAGAGTAGGGAACTGCCAGGCATCAAATAAAACGAAAGGCTCAGTCGAAAGACTGGGCCTTGCATCAATCTCCAATTATCACTTGACTCTATAGTGACTATAGAGATTTTAATGGAGGCTGAATAGAAGATTTTCAGGAGTTACTCTCTAGA | *Klebsiella pneumoniae* CG43 plasmid pLVPK |
| pCON-MP-DV  (*Bgl*II/*Xba*I) | AGATCTCTACCCAGATGTTTGACTGTTCGTGGCACTTTCACCATGGCAATTGCCCAACCCTTGCAAAATGCCGCACGCCTCTACAGATCGAGAGCCAGAACACTTCTCGCGCAAATCAACCAAGTGCCGTTTTAACTGCAACAGCGCGGACACACGCATTTCCACCTGTTGAATATGGGCCTCCAGCAGCGTGATGACCTCCCCACAGTCCTGCATCGGGTTGTCTCGCAGACCCAGCAATGCGCGAATCTCGCTCAACGTCATGTCGAGCGAACGGCAATGACGGATGAATTGCAAGCGCTCAATGTGCGCCTCACCGTACAAGCGGATGCTGCCGTAAGGCTTGTCCGGTTCCCGCAACAGGCCCTTGCGCTGATAGAAGCGGATTGTCTCCACGTTGACCCCGGCCGCCTTGGCAAAAACGCCAATGGTCAGGTTTTCCAAATTATTTTCCATCGTTCGCGCAACGCCTCCTGGTACCTGTGCTTTGTTAAAGGGTTTCAACAGATATTCAAATCGCTTGACTCCGTACATGAGTACGGAAGTAAGGTTACGCTATCCAATCCAAATTCAAAAGGGCCAACGTTCTAGA | Chimeric MerR-PbrR regulator |
| pNAT-MP-DV  (*Bgl*II/*Xba*I) | AGATCTCTACCCAGATGTTTGACTGTTCGTGGCACTTTCACCATGGCAATTGCCCAACCCTTGCAAAATGCCGCACGCCTCTACAGATCGAGAGCCAGAACACTTCTCGCGCAAATCAACCAAGTGCCGTTTTAACTGCAACAGCGCGGACACACGCATTTCCACCTGTTGAATATGGGCCTCCAGCAGCGTGATGACCTCCCCACAGTCCTGCATCGGGTTGTCTCGCAGACCCAGCAATGCGCGAATCTCGCTCAACGTCATGTCGAGCGAACGGCAATGACGGATGAATTGCAAGCGCTCAATGTGCGCCTCACCGTACAAGCGGATGCTGCCGTAAGGCTTGTCCGGTTCCCGCAACAGGCCCTTGCGCTGATAGAAGCGGATTGTCTCCACGTTGACCCCGGCCGCCTTGGCAAAAACGCCAATGGTCAGGTTTTCCAAATTATTTTCCATATCGCTTGACTCCGTACATGAGTACGGAAGTAAGGTTACGCTATCCAATCCAAATTCAAAAGGGCCAACGTTCTAGA | Chimeric MerR-PbrR regulator |
| pKp-2P-DV  (*Bgl*II/*Xba*I) | AGATCTTTACCCAGATGTTTGACTGTTCGTGGCACTTTCACCATGGCAATTGCCCAACCCTTGCAAAATGCCGCACGCCTCTACAGATCGAGAGCCAGAACACTTCTCGCGCAAATCAACCAAGTGCCGTTTTAACTGCAACAGCGCGGACACACGCATTTCCACCTGTTGAATATGGGCCTCCAGCAGCGTGATGACCTCCCCACAGTCCTGCATCGGGTTGTCTCGCAGACCCAGCAATGCGCGAATCTCGCTCAACGTCATGTCGAGCGAACGGCAATGACGGATGAATTGCAAGCGCTCAATGTGCGCCTCACCGTACAACCGAAAGTTGCCACCGCTTCGCGCTGGCTTTGGCAGTAGCCCTTCCTTCTCGTAGTAGCGGATGGTCACGACCTCGCACCCAGAGCGCTTGGCGAGGTCGCCAATTCTGATTTCCATCGTTCGCGCAACGCCTCCTGGTACCTGTGCTTTGTTAAAGGGTTTCAACAGATATTCAATTACCCAGATGTTTGACTGTTCGTGGCACTTTCACCATGGCAATTGCCCAACCCTTGCAAAATGCCGCACGCCTCTACAGATCGAGAGCCAGAACACTTCTCGCGCAAATCAACCAAGTGCCGTTTTAACTGCAACAGCGCGGACACACGCATTTCCACCTGTTGAATATGGGCCTCCAGCAGCGTGATGACCTCCCCACAGTCCTGCATCGGGTTGTCTCGCAGACCCAGCAATGCGCGAATCTCGCTCAACGTCATGTCGAGCGAACGGCAATGACGGATGAATTGCAAGCGCTCAATGTGCGCCTCACCGTACAACCGAAAGTTGCCACCGCTTCGCGCTGGCTTTGGCAGTAGCCCTTCCTTCTCGTAGTAGCGGATGGTCACGACCTCGCACCCAGAGCGCTTGGCGAGGTCGCCAATTCTGATTTCCATGCATCAATCTCCAATTATCACTTGACTCTATAGTGACTATAGAGATTTTAATGGAGGCTGAATAGAAGATTTTCAGGAGTTACTCTCTAGA | *Klebsiella pneumoniae* CG43 plasmid pLVPK |

The constitutive promoter P*_cueR_* is shown in a turquoise background. The constitutive promoter P302, P406, P479, P535, P637, P699, and PJ23119 are shown in a yellow background (the ribosome site is lowercase). The *rrn*B terminator is shown in a purple background. The ORFs of various PbrR homologs are shown in green. The amino acid sequences of MerR are shown in blue. The start codon of metalloregulator is shown in a green background and the end codon is shown in a red background. Restriction sites are underlined. The divergent pbr promoter is shown in orange. The divergent mer promoter is shown in a grey background.

**Table. S2 Summary of characterized PbrR homologs**

| Construct | PbrR Homologs | Detection Range (µM) | Specificity | LOD (µM) |
| --- | --- | --- | --- | --- |
| pCm-DV | PbrR.Cm | 0.0977 - 50 | Cd(II), Pb(II) | 0.0977 |
| pCmc-DV | PbrR.Cmc | ND | Pb(II) and Zn(II) | ND |
| pPa-DV | PbrR.Pa | 0.0061-50 | Pb(II), Cd(II) and Hg(II) | 0.0061 |
| pKp-DV | PbrR.Kp | 0.0061-50 | Pb(II), Hg(II) | 0.0008 |

**ND: not determined.**


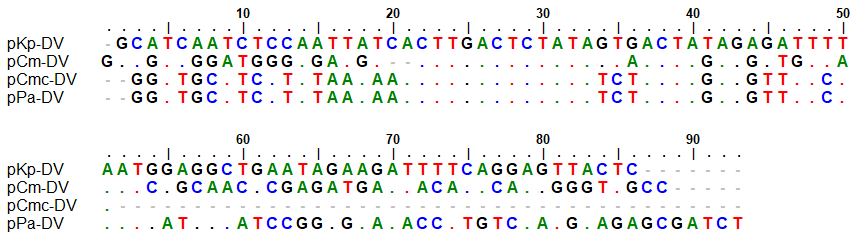


**Figure S1.** Sequence alignment of the *pbrR*-*pbrA*/*cadA* intergenic region in four *pbr* operon homologs.

| ATGGAAAATAATTTGGAAAACCTGACCATTGGCGTTTTTGCCAAGGCGGCCGGGGTCAACGTGGAGACAATCCGCTTCTATCAGCGCAAGGGCCTGTTGCGGGAACCGGACAAGCCTTACGGCAGCATCCGCTTGTACGGTGAGGCGCACATTGAGCGCTTGCAATTCATCCGTCATTGCCGTTCGCTCGACATGACGTTGAGCGAGATTCGCGCATTGCTGGGTCTGCGAGACAACCCGATGCAGGACTGTGGGGAGGTCATCACGCTGCTGGAGGCCCATATTCAACAGGTGGAAATGCGTGTGTCCGCGCTGTTGCAGTTAAAACGGCACTTGGTTGATTTGCGCGAGAAGTGTTCTGGCTCTCGATCTGTAGAGGCGTGCGGCATTTTGCAAGGGTTGGGCAATTGCCATGGTGAAAGTGCCACGAACAGTCAAACATCTGGGTAG |
| --- |
| MENNLENLTIGVFAKAAGVNVETIRFYQRKGLLREPDKPYGSIRLYGEAHIERLQFIRHCRSLDMTLSEIRALLGLRDNPMQDCGEVITLLEAHIQQVEMRVSALLQLKRHLVDLREKCSGSRSVEACGILQGLGNCHGESATNSQTSG* |

**Figure S2.** Sequence of the chimeric PbrRMerR. The metal-binding domain (MBD) of PbrR is shown in blue, and the DNA-binding domain (DBD) of MerR is shown in green.
